# Fluoropyrimidine with or without platinum as first-line chemotherapy in patients with advanced gastric cancer and severe peritoneal metastasis: a multicenter retrospective study

**DOI:** 10.1186/s12885-019-5720-3

**Published:** 2019-07-03

**Authors:** Hiroyuki Arai, Satoru Iwasa, Narikazu Boku, Masahiro Kawahira, Hirofumi Yasui, Toshiki Masuishi, Kei Muro, Keiko Minashi, Shuichi Hironaka, Naoki Fukuda, Daisuke Takahari, Takako Eguchi Nakajima

**Affiliations:** 1 Department of Clinical Oncology, St. Marianna University School of Medicine, Kawasaki, Japan; 2 Department of Gastrointestinal Medical Oncology, National Cancer Center Hospital, Tokyo, Japan; 3 Division of Gastrointestinal Oncology, Shizuoka Cancer Center, Shizuoka, Japan; 4 Department of Clinical Oncology, Aichi Cancer Center Hospital, Nagoya, Japan; 5 Clinical Trial Promotion Department, Chiba Cancer Center, Chiba, Japan; 6 Department of Gastroenterology, Cancer Institute Hospital of the Japanese Foundation for Cancer Research, Tokyo, Japan

**Keywords:** Gastric cancer, Peritoneal metastasis, Chemotherapy, Massive ascites, Inadequate oral intake

## Abstract

**Background:**

There is no standard first-line chemotherapy for advanced gastric cancer with severe peritoneal metastasis. Although fluoropyrimidine is often used, its efficacy is limited, and it remains unclear whether combination therapy with platinum improves clinical outcomes.

**Methods:**

This retrospective study involved patients at six Japanese academic hospitals between 2010 and 2016. Patients with advanced gastric cancer and severe peritoneal metastasis were included if they had massive ascites and/or inadequate oral intake requiring intravenous nutritional support. We then compared the efficacy and safety of fluoropyrimidine monotherapy with those of fluoropyrimidine/platinum combination therapy.

**Results:**

Compared with the combination therapy group (*n* = 64), the monotherapy group (*n* = 65) had worse general health (more patients with elderly age, performance status > 2, and having both massive ascites and inadequate oral intake). Both overall survival (9.0 vs 5.0 months, *p* < 0.01) and progression-free survival (4.3 vs 2.3 months, *p* < 0.01) were significantly longer in the combination group, and the significance remained after adjusting for prognostic variables (hazard ratios of 0.47 and 0.41, respectively; *p* < 0.01). Improvements in ascites and oral intake were also greater in the combination group. Although neutropenia (grade ≥ 3) occurred more frequently with combination therapy, both treatments in this study were tolerable.

**Conclusions:**

Combination therapy with fluoropyrimidine and platinum might be more effective than monotherapy with fluoropyrimidine and was tolerable for patients with advanced gastric cancer and severe peritoneal metastasis.

## Background

Gastric cancer (GC) is the fifth most common cancer and the third leading cause of cancer-related death worldwide [1]. The standard first-line chemotherapy for patients with erb-b2 receptor tyrosine kinase 2 (ERBB2, also known as HER2)-negative advanced GC (AGC) in Japan is a combination therapy with fluoropyrimidine plus platinum (e.g., S-1 or capecitabine plus cisplatin or oxaliplatin) [2–4].

Peritoneal metastasis is very common in AGC and causes a range of serious clinical complications, including massive ascites, bowel obstruction, jaundice, and hydronephrosis, that worsen prognosis and quality of life [5, 6]. When massive ascites is present, intolerable abdominal fullness often makes it difficult to administer cisplatin which requires adequate hydration. Subacute or acute bowel obstruction causing nausea, vomiting, and malabsorption, also makes stable oral fluoropyrimidine intake impractical. For these reasons, patients with severe peritoneal metastasis (SPM) associated with massive ascites and/or inadequate oral intake were excluded from pivotal phase III trials [2–4, 7]. Even in a phase III trial (Japan Clinical Oncology Group [JCOG] 0106), comparing fluorouracil (5-FU) by continuous infusion (ci) with 5-FU plus methotrexate (MTX) in AGC patients with peritoneal metastasis, patients with massive ascites were excluded and only a few patients (14% [32/237]) with inadequate oral intake were included [8]. Based on such backgrounds, no standard first-line chemotherapy has been established and an unmet need exists for prolongation the survival time in AGC patients with SPM. On the other hand, adaptation of patients to chemotherapy should be carefully judged before treatment initiation because an aggressive disease course can immediately change the condition of patients into a non-adaptive state to chemotherapy.

In clinical practice, fluoropyrimidine monotherapy regimens, such as options including 5-FU plus *l*-leucovorin (*l*-LV), 5-FU ci, and 5-FU plus MTX, are generally used as first-line chemotherapy for patients with AGC and SPM [9, 10]. However, retrospective studies have shown that fluoropyrimidine monotherapy has modest efficacy and feasible toxicity with median survival times of only 4.6–6.0 months, indicating that further investigation is warranted [9, 10]. One option is to use platinum in combination with fluoropyrimidine [11]. Here, oxaliplatin is a third-generation platinum agent and suitable even for patients with massive ascites because hydration is needless. Recently, oxaliplatin was approved for the treatment of GC in Japan based on its non-inferiority to cisplatin in phase III trials of patients with AGC without SPM [4, 12].

The lack of prospective data in patients with AGC and SPM makes it unclear whether combination therapy with fluoropyrimidine plus platinum could be more effective than fluoropyrimidine monotherapy in patients with AGC and SPM. Therefore, we investigated the efficacy and safety of fluoropyrimidine/platinum combination therapy compared with fluoropyrimidine monotherapy when used as the first-line chemotherapy in patients with AGC and SPM.

## Methods

### Study design

This was a retrospective study of patients with AGC and SPM who received first-line chemotherapy between July 2010 and September 2016 at six institutions in Japan. We compared the efficacy and safety of fluoropyrimidine/platinum combination therapy (the FP group) with those of fluoropyrimidine monotherapy (the F group). All patient data were extracted from a database at each center. This study was approved by the institutional review board in each center. Written informed consent was obtained from all patients before treatment initiation.

### Eligibility criteria and definitions

All the patients in this study had SPM. SPM was defined as peritoneal metastasis associated with massive ascites and/or inadequate oral intake, with the latter defined as requiring intravenous nutritional support. The degree of ascites was evaluated by computed tomography and classified as follows: “none” if undetectable; “mild” if localized to the pelvic cavity or upper abdominal cavity; “moderate” if inconsistent with either mild or massive ascites; and “massive” if extending continuously between the pelvic cavity and upper abdominal cavity. The eligibility criteria for this study were as follows: (1) histologically proven adenocarcinoma of the stomach or gastroesophageal junction; (2) HER2-negative or unknown tumor; (3) having SPM; (4) absence of concomitant advanced malignant disease; and (5) if the patient had recurrent disease, the recurrence occurred at least 6 months after the last dose of adjuvant chemotherapy. We excluded patients who had participated in the randomized phase II/III trial of 5-FU/*l*-LV compared with 5-FU/*l*-LV plus paclitaxel for AGC with SPM (JCOG1108/WJOG7312G trial: UMIN000010949) because the trial was ongoing and not available for analysis at start of this study. We also excluded patients who had inadequate hepatic and/or renal function (serum total bilirubin > 3.0 mg/dl, serum creatinine > 2.0 mg/dl).

### Chemotherapy

The F group received any fluoropyrimidine monotherapy regimen with or without chemical modulators (e.g., *l*-LV or MTX). The FP group received cisplatin or oxaliplatin in combination with any fluoropyrimidine. If a 5-FU regimen was modified to an S-1 regimen due to improved oral intake, the S-1 regimen was regarded as second-line chemotherapy.

### Assessment of response in ascites and improvement of Oral intake

We compared the degree of ascites between baseline and during treatment, and determined the best response in ascites as follows: “complete response (CR)” if the ascites completely disappeared; “partial response (PR)” if there was a decrease by at least one degree from baseline; “stable disease” if there was no change from baseline; “progressive disease” if there was an increase by at least one degree from baseline; and “not evaluated” if it was impossible to evaluate because ascites was drained before assessment or because there was no recorded assessment. We defined the response rate in ascites as the proportion of patients with the best CR or PR among those with ascites at baseline.

The improvement rate of oral intake was also defined as the proportion of patients whose oral intake recovered and did not require nutritional support for at least 7 days among the patients who had inadequate oral intake at baseline.

### Statistical analysis

We compared patient demographics between the FP and F groups. *P* values were estimated by chi-square tests for categorical variables and by Mann–Whitney *U* tests for continuous variables. Time to treatment failure (TTF) was defined as the time from treatment start to the last dose of chemotherapy. Overall survival (OS) was defined as the time from treatment start to death from any cause, and progression-free survival (PFS) was defined as the time from treatment start to disease progression or death from any cause. Patients who had no events during the observation period were censored at the last follow-up date (cut-off date, June 30, 2017).

Both OS and PFS were estimated by the Kaplan–Meier method and compared between the groups by the log-rank test. Hazard ratios (HRs) and confidence intervals (CIs) were then estimated using Cox proportional hazards models in both univariate and multivariate analyses. Covariates with a *p*-value < 0.20 in the univariate analysis were included into the multivariate analysis, and the Eastern Cooperative Oncology Group performance status (PS; i.e., 0–1 or 2–4) and SPM subtype (i.e., massive ascites, inadequate oral intake, or both) were also included because these covariates were considered clinically important. The response rate in ascites, the improvement rate of oral intake, and the safety of each regimen were compared by chi-square tests.

We also identified patients who might not be adaptive to any type of chemotherapy to prevent such patients from undergoing chemotherapy as they would not benefit from it. To this end, we considered patients who died within 90 days after treatment initiation to be potentially “non-adaptive” and survivors to be “adaptive.” Further, we compared characteristics between the “non-adaptive” and “adaptive” patients and conducted univariate analysis to estimate odds ratio (OR) of each risk factor for early death, i.e., death within 90 days after treatment initiation, by logistic regression. In this analysis, we used the median value of all patients as the cut-off value, e.g., serum albumin (3.1 g/ml) and CRP (2.2 mg/dl) levels.

All analyses were two-sided, and a *p*-value < 0.05 was considered statistically significant. All statistical analyses were performed using StatView ver 5.0 software (SAS Institute, Cary, NC, USA).

## Results

### Patients

After excluding 10 ineligible patients (6 participated in JCOG1108/WJOG7312G trial, 3 had inadequate hepatic function, and 1 had inadequate renal function) from the available cohort, we finally recruited 129 patients (64 in the FP group and 65 in the F group). The baseline characteristics of the participants are shown in Table 1. Overall, 40% had a PS > 2 (including 7 patients with PS 3 and 1 patient with PS 4), and the median (range) serum albumin level was 3.1 (1.8–4.4) g/ml. The F group tended to be older (median, 67 vs 62 years) and include more patients with a PS > 2 (51% vs 30%), fewer patients with massive ascites only (35% vs 61%), and more patients with both massive ascites and inadequate oral intake (35% vs 14%). Median serum albumin and C-reactive protein levels were comparable between both groups.

**Table 1:** Patient characteristics

Characteristics	All patients	FP group	F group	*p*-value
	(*n* = 129)	(*n* = 64)	(n = 65)	
	No. (%)	No. (%)	No. (%)	
Age (years)				
Median (range)	65 (24–94)	62 (27–94)	67 (24–89)	0.02
< 65	61 (47)	36 (56)	25 (38)	0.04
> 65	68 (53)	28 (44)	40 (62)	
Sex				
Male	68 (53)	33 (52)	35 (54)	0.80
Female	61 (47)	31 (48)	30 (46)	
ECOG PS				
0	9 (7)	4 (6)	5 (8)	
1	68 (53)	41 (64)	27 (42)	
2	44 (34)	16 (25)	28 (43)	
3	7 (5)	2 (3)	5 (8)	
4	1 (1)	1 (2)	0 (0)	
0–1	77 (60)	45 (70)	32 (49)	0.01
2–4	52 (40)	19 (30)	33 (51)	
Histology				
Intestinal	22 (17)	10 (16)	12 (18)	0.81
Diffuse	102 (79)	52 (81)	50 (77)	
Unknown	5 (4)	2 (3)	3 (5)	
Disease status				
Advanced	120 (93)	58 (91)	62 (95)	0.29
Recurrent	9 (7)	6 (9)	3 (5)	
Primary tumor				
Presence	113 (88)	54 (84)	59 (91)	0.27
Absence	16 (12)	10 (16)	6 (9)	
Primary site				
Stomach	123 (95)	59 (92)	64 (98)	0.09
GEJ	6 (5)	5 (8)	1 (2)	
No. of metastatic sites				
1–2	100 (78)	49 (77)	51 (78)	0.80
> 3	29 (22)	15 (23)	14 (22)	
Target lesion				
Presence	56 (43)	24 (38)	32 (49)	0.18
Absence	73 (57)	40 (62)	33 (51)	
Subtype of SPM				
Massive ascites	62 (48)	39 (61)	23 (35)	0.01
Inadequate oral intake	35 (27)	16 (25)	19 (29)	
Both	32 (25)	9 (14)	23 (35)	
Serum albumin level (g/ml)				
Median (range)	3.1 (1.8–4.4)	3.1 (1.8–4.3)	3.1 (1.8–4.4)	0.87
Serum CRP level (mg/dl)				
Median (range)	2.2 (0.0–24.0)	1.8 (0.1–20.7)	2.6 (0.0–24.0)	0.86

*CRP* C-reactive protein, *ECOG PS*: Eastern Cooperative Oncology Group performance status, *GEJ* gastroesophageal junction, *SPM* severe peritoneal metastasis

### Chemotherapy exposure

**Fig. 1:**
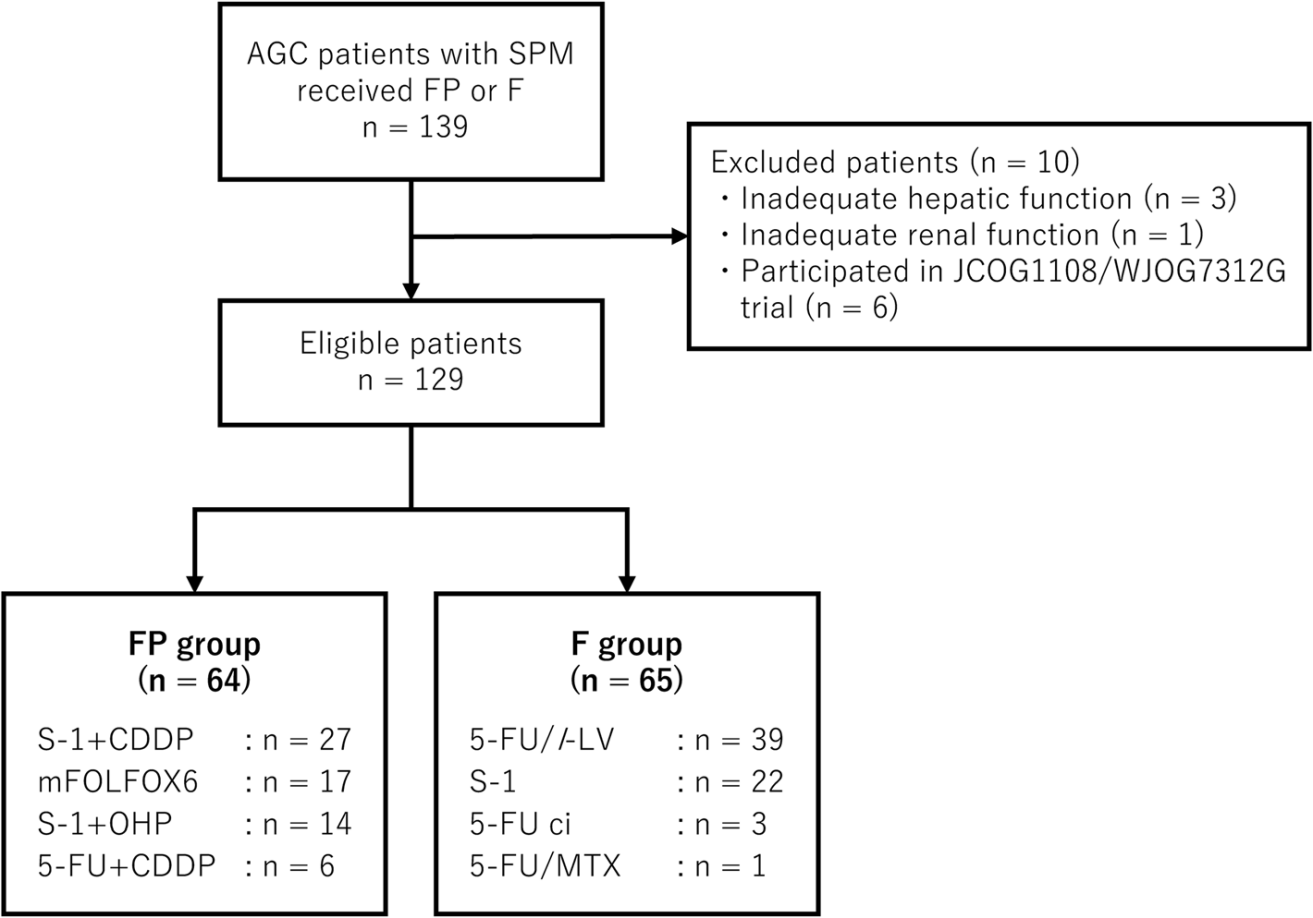
CONSORT diagram. Abbreviation: AGC, advanced gastric cancer; CDDP, cisplatin; ci, continuous infusion; F, fluoropyrimidine; FP, fluoropyrimidine plus platinum; LV, leucovorin; MTX, methotrexate; OHP, oxaliplatin; SPM, severe peritoneal metastasis

S-1 plus cisplatin (*n* = 27), modified FOLFOX6 (*n* = 17), S-1 plus oxaliplatin (*n* = 14), and 5-FU plus cisplatin (*n* = 6) were used in the FP group. By contrast, 5-FU/*l*-LV (*n* = 39), S-1 (*n* = 22), 5-FU ci (*n* = 3), and 5-FU/MTX (*n* = 1) were used in the F group (Fig. 1). The dosage and treatment schedules of each regimen were similar to those previously reported [2, 4, 8, 13, 14].

The first-line treatment was discontinued in all patients at median TTFs of 3.3 and 1.4 months in the FP and F groups, respectively. Second-line chemotherapy was given to 46 patients (72%) in the FP group and 33 patients (51%) in the F group (*p* = 0.01). Most patients in both the FP group (33/46; 72%) and the F group (25/33; 76%) received taxane-based chemotherapy.

### Reasons for discontinuation

Reasons for treatment discontinuation in the FP group were disease progression (78% [50/64]), adverse events (6% [4/64]), change from 5-FU to S-1 regimens because of improved oral intake (2% [1/64]), withdrawal of consent (2% [1/64]), and other reasons (13% [8/64]); the adverse events leading to treatment discontinuation in the FP group were peripheral neuropathy (*n* = 2), fatigue (*n* = 1), and stomatitis (*n* = 1). Reasons for treatment discontinuation in the F group were disease progression (77% [50/65]), adverse events (9% [6/65]), change from 5-FU to S-1 regimens because of improved oral intake (11% [7/65]), and other reasons (3% [2/65]); the adverse events leading to treatment discontinuation in the F group were sepsis (*n* = 2), febrile neutropenia (*n* = 1), appetite loss (*n* = 1), stomatitis (*n* = 1), and angina (*n* = 1).

### Efficacy

**Fig. 2:**
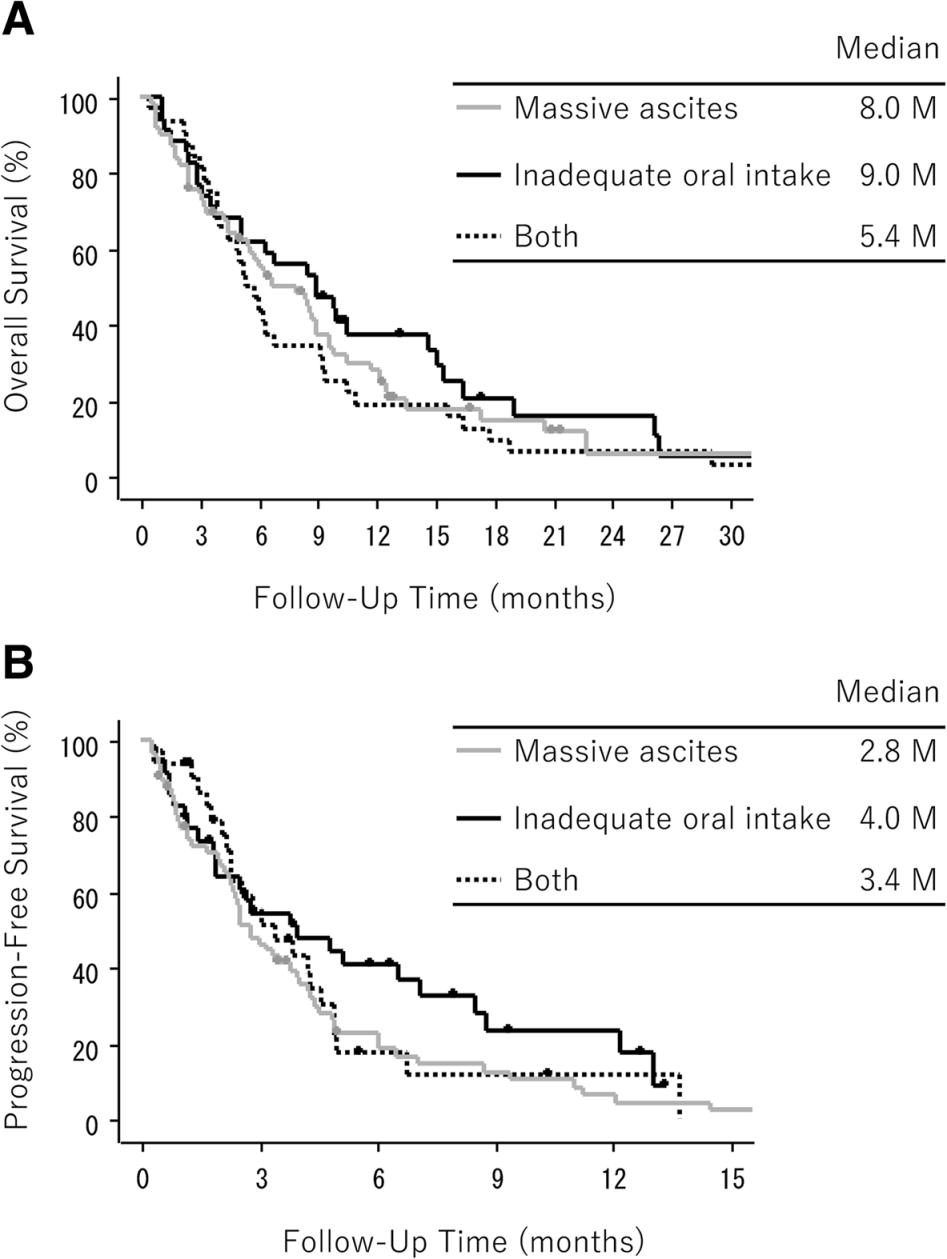
Kaplan–Meier curves of overall survival (A) and progression-free survival (B) according to the subtype of severe peritoneal metastasis

The number of OS events was 50 (78%) in the FP group and 61 (94%) in the F group. Totally, all the patients showed median OS and PFS of 6.7 and 3.1 months, respectively. Analysis by SPM subtypes revealed OS for patients with massive ascites only, inadequate oral intake only, and both of 8.0, 9.0, and 5.4 months, respectively, with corresponding PFS times of 2.8, 4.0, and 3.4 months, respectively (Fig. 2).

**Fig. 3:**
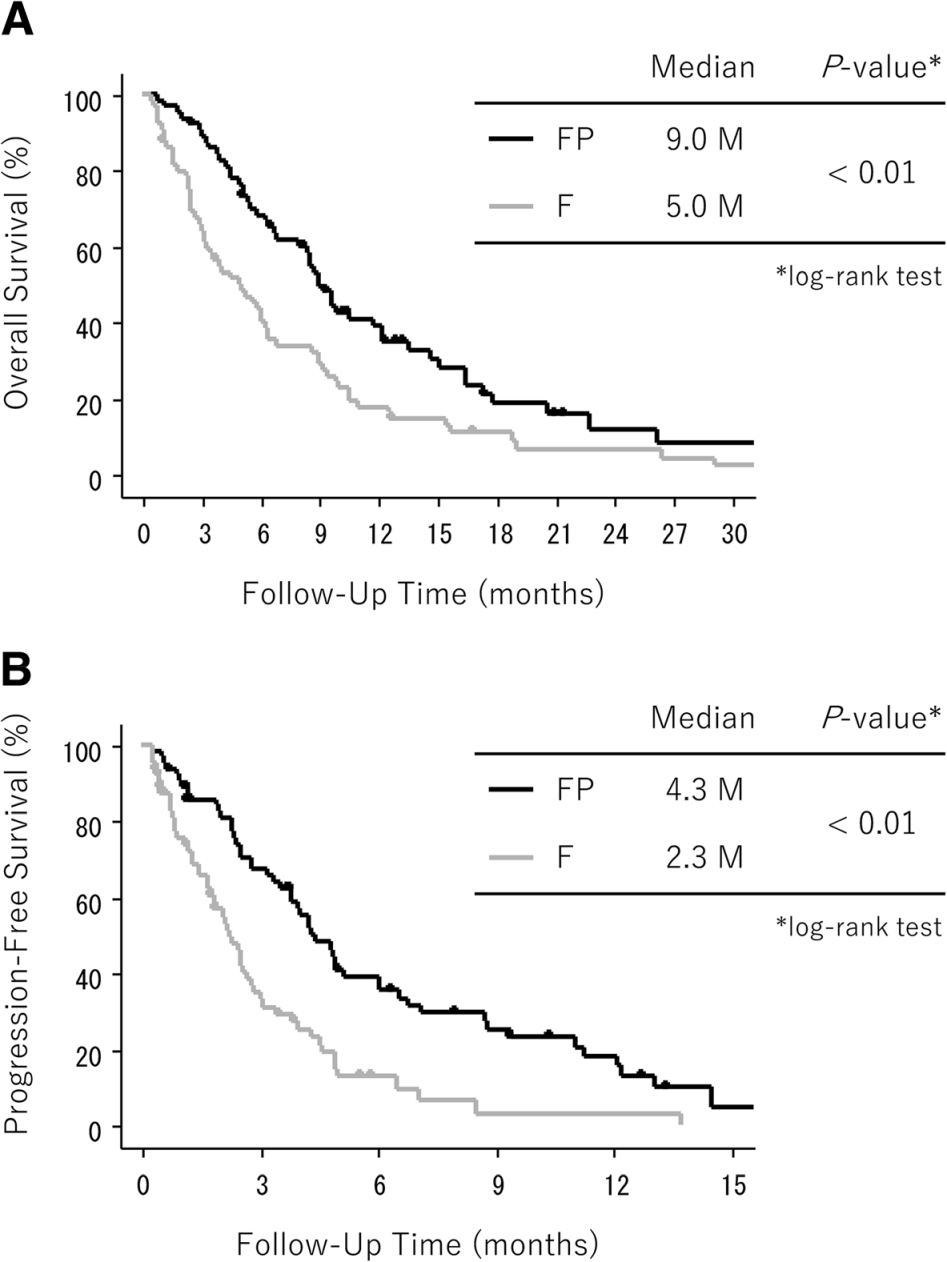
Kaplan–Meier curves of overall survival (**a**) and progression-free survival (**b**) between the FP and F groups. Abbreviation: F, fluoropyrimidine; FP, fluoropyrimidine plus platinum

The median OS was 9.0 months in the FP group and 5.0 months in the F group, with a statistical significance (HR, 0.56; 95% CI, 0.39–0.82; log-rank *p* < 0.01) (Fig. 3). Univariate analysis revealed that intestinal histological type, recurrent disease, ≤ 2 metastatic sites, and serum albumin ≥3.1 g/ml were predictors of better OS (*p* < 0.20). After adjustment for these factors, PS and the SPM subtype, OS in the FP group remained superior to that in the F group (HR, 0.47; 95% CI, 0.31–0.72; *p* < 0.01) (Table 2).

**Table 2:** Univariate and multivariate analyses of the prognostic factors of OS in AGC patients with SPM (*n* = 129)

Variables	Univariate analysis			Multivariate analysis		
	HR	[95% CI]	*p*-value	HR	[95% CI]	*p*-value
Regimen						
FP (vs. F)	0.56	[0.39–0.82]	< 0.01	0.47	[0.31–0.72]	< 0.01
Age						
≥65 (vs. < 65)	0.80	[0.55–1.17]	0.25			
Sex						
Female (vs. male)	1.05	[0.72–1.54]	0.78			
PS						
2–4 (vs. 0–1)	1.22	[0.83–1.79]	0.32	0.95	[0.62–1.48]	0.83
Histology						
Intestinal (vs. diffuse)	0.71	[0.43–1.19]	0.19	0.70	[0.41–1.19]	0.18
Unknown (vs. diffuse)	NA		NA	NA		NA
Disease status						
Recurrent (vs. advanced)	0.58	[0.25–1.32]	0.19	0.86	[0.36–2.10]	0.75
Primary site						
GEJ (vs. stomach)	0.98	[0.40–2.41]	0.96			
No. of metastatic sites						
≥3 (vs. 1–2)	1.43	[0.92–2.23]	0.11	1.64	[0.97–2.76]	0.07
Target lesion						
Absence (vs. presence)	0.95	[0.65–1.38]	0.78			
Subtype of SPM						
Inadequate oral intake (vs. massive ascites)	0.81	[0.52–1.29]	0.38	0.58	[0.33–1.00]	0.05
Both (vs. massive ascites)	1.09	[0.69–1.71]	0.71	0.76	[0.44–1.29]	0.31
Serum albumin level						
< 3.1 g/ml (vs. > 3.1 g/ml)	1.42	[0.97–2.07]	0.07	1.43	[0.94–2.17]	0.09

*AGC* advanced gastric cancer, *CI* confidence interval, *ECOG PS* Eastern Cooperative Oncology Group performance status, *GEJ* gastroesophageal junction, *HR* hazard ratio, *NA* not assessed, *OS* overall survival, *SPM* severe peritoneal metastasis

PFS was also significantly longer in the FP group than in the F group (median, 4.3 vs 2.3 months; HR, 0.44; 95% CI, 0.30–0.66; log-rank *p* < 0.01) (Fig. 3). Univariate analysis identified the recurrent disease and having only inadequate oral intake as predictors of better PFS (*p* < 0.20). After adjustment for these factors and PS, PFS in the FP group remained superior to that in the F group (HR, 0.41; 95% CI, 0.26–0.64; *p* < 0.01) (Table 3).

**Table 3:** Univariate and multivariate analyses of the prognostic factors of PFS in AGC patients with SPM (*n* = 129)

Variables	Univariate analysis			Multivariate analysis		
	HR	[95% CI]	*p*-value	HR	[95% CI]	*p*-value
Regimen						
FP (vs. F)	0.44	[0.30–0.66]	< 0.01	0.41	[0.26–0.64]	< 0.01
Age						
≥65 (vs. < 65)	0.99	[0.67–1.45]	0.95			
Sex						
Female (vs. male)	0.96	[0.65–1.41]	0.82			
ECOG PS						
2–4 (vs. 0–1)	1.28	[0.86–1.90]	0.22	1.04	[0.68–1.60]	0.86
Histology						
Intestinal (vs. diffuse)	0.81	[0.48–1.39]	0.45			
Unknown (vs. diffuse)	NA		NA			
Disease status						
Recurrent (vs. advanced)	0.48	[0.20–1.19]	0.11	0.60	[0.24–1.50]	0.27
Primary site						
GEJ (vs. stomach)	0.55	[0.20–1.53]	0.25			
No. of metastatic sites						
≥3 (vs. 1–2)	1.28	[0.86–1.90]	0.22			
Target lesion						
Absence (vs. presence)	1.03	[0.69–1.53]	0.90			
Subtype of SPM						
Inadequate oral intake (vs. massive ascites)	0.66	[0.41–1.07]	0.09	0.64	[0.40–1.04]	0.07
Both (vs. massive ascites)	0.88	[0.54–1.44]	0.61	0.57	[0.34–0.96]	0.03
Serum albumin level						
< 3.1 g/ml (vs. > 3.1 g/ml)	1.10	[0.74–1.62]	0.65			

*AGC* advanced gastric cancer, *CI* confidence interval, *ECOG PS* Eastern Cooperative Oncology Group performance status, *GEJ* gastroesophageal junction, *HR* hazard ratio, *NA* not assessed, *PFS* progression-free survival, *SPM* severe peritoneal metastasis

Response rate in ascites was 51% (30/59) in the FP group and 17% (10/60) in the F group (*p* < 0.01). Improvement rate of oral intake was 64% (16/25) in the FP group and 43% (18/42) in the F group (*p* = 0.09).

### Safety

The incidence of all grades of adverse events was significantly higher in the FP group for leukopenia (63% vs 35%), neutropenia (61% vs 26%), anemia (80% vs 57%), thrombocytopenia (58% vs 14%), and sensory neuropathy (38% vs 0%), compared with the F group. Regarding the incidences of grade 3 or 4 adverse events, only neutropenia was significantly higher in the FP group (36% vs 11%), whereas the others were similar (Table 4). There were no treatment-related deaths. However, we observed death within 30 days form the last dose of chemotherapy in 23 patients (18%) totally: 7 (11%) in the FP group and 16 (25%) in the F group. All of these patients were died from disease progression. This included 7 patients (5%) who died after the first administration of chemotherapy: 1 (2%) in the FP group and 6 (9%) in the F group.

**Table 4:** Adverse Events

Adverse Event	FP (*n* = 64)		F (*n* = 65)		*p*-value	
	All Gr.	Gr. 3–4	All Gr.	Gr. 3–4		
	No. (%)	No. (%)	No. (%)	No. (%)	All Gr.	Gr. 3–4
Leukocytopenia	40 (63)	11 (17)	23 (35)	5 (8)	< 0.01	0.10
Neutropenia	39 (61)	23 (36)	17 (26)	7 (11)	< 0.01	< 0.01
Anemia	51 (80)	12 (19)	37 (57)	15 (23)	< 0.01	0.55
Thrombocytopenia	37 (58)	3 (5)	9 (14)	0 (0)	< 0.01	0.08
Febrile neutropenia	3 (5)	3 (5)	3 (5)	3 (5)	0.98	0.98
Anorexia	42 (66)	10 (16)	37 (57)	17 (26)	0.31	0.14
Nausea	40 (63)	3 (5)	34 (52)	1 (2)	0.24	0.30
Vomiting	18 (28)	1 (2)	23 (35)	2 (3)	0.38	0.57
Diarrhea	16 (25)	3 (5)	18 (28)	1 (2)	0.73	0.30
Stomatitis	14 (22)	1 (2)	7 (11)	0 (0)	0.09	0.31
Fatigue	27 (42)	2 (3)	27 (42)	3 (5)	0.94	0.66
Sensory neuropathy	24 (38)	1 (2)	0 (0)	0 (0)	< 0.01	0.31

### Risk factors for early death

We observed early death in 28 patients (22% of 129 patients): 7 patients (11%) in the FP group and 21 patients (32%) in the F group. These 28 patients were deemed to be potentially “non-adaptive” to any type of chemotherapy, whereas the remaining 101 patients were deemed to be “adaptive.” “Non-adaptive” patients had significantly higher serum CRP level than “adaptive” patients (Table 5). Univariate analysis showed two significant risk factors for early death: serum albumin level < 3.1 g/ml (OR, 2.43; 95% CI, 1.02–5.78; *p* = 0.05) and serum CRP level > 2.2 mg/dl (OR, 2.87; 95% CI, 1.16–7.13; *p* = 0.02) (Table 6).

**Table 5:** Characteristics of “adaptive” or “non-adaptive” patients

Characteristics	Adaptive patients	Non-adaptive patients	*p*-value
	(*n* = 101)	(*n* = 28)	
	No. (%)	No. (%)	
Age (years)			
Median (range)	65 (24–86)	67 (34–94)	0.09
Sex			
Male	51 (50)	17 (61)	0.34
Female	50 (50)	11 (39)	
ECOG PS			
0	8 (8)	1 (4)	
1	56 (55)	12 (43)	
2	32 (32)	12 (43)	
3	4 (4)	3 (11)	
4	1 (1)	0 (0)	
0–1	64 (63)	13 (46)	0.11
2–4	37 (37)	15 (54)	
Disease status			
Advanced	92 (91)	28 (100)	0.10
Recurrent	9 (9)	0 (0)	
Primary site			
Stomach	97 (96)	26 (93)	0.48
GEJ	4 (4)	2 (7)	
No. of metastatic sites			
1–2	81 (80)	19 (68)	0.17
> 3	20 (20)	9 (32)	
Subtype of SPM			
Massive ascites	46 (45)	16 (57)	0.50
Inadequate oral intake	28 (27)	7 (25)	
Both	27 (27)	5 (18)	
Serum albumin level (g/ml)			
Median (range)	3.1 (1.8–4.4)	2.9 (2.0–4.2)	0.13
Serum CRP level (mg/dl)			
Median (range)	1.6 (0.0–20.7)	4.8 (0.1–24.0)	< 0.01

Patients who died within 90 days after treatment initiation were defined as “non-adaptive”

Other patients were defined as “adaptive”

*CRP* C-reactive protein, *ECOG PS* Eastern Cooperative Oncology Group performance status, *GEJ* gastroesophageal junction, *SPM* severe peritoneal metastasis

**Table 6:** Univariate analysis for risk factors for early death (within 90 days after treatment initiation)

Variable	Univariate analysis			
	N	OR	[95% CI]	*p*-value
Age (years)				
< 65	11/61	1.00		
≥65	17/68	1.52	[0.65–3.56]	0.34
Sex				
Male	17/68	1.00		
Female	11/61	0.66	[0.28–1.55]	0.34
ECOG PS				
0–1	13/77	1.00		
2–4	15/52	2.00	[0.86–4.65]	0.11
Disease status				
Advanced	28/120	NC		NC
Recurrent	0/9			
Primary site				
Stomach	26/123	1.00		
GEJ (vs. stomach)	2/6	1.87	[0.32–10.76]	0.49
No. of metastatic sites				
1–2	19/100	1.00		
≥3	9/29	1.92	[0.76–4.87]	0.17
Subtype of SPM				
Massive ascites	16/62	1.00		
Inadequate oral intake	7/35	0.72	[0.26–1.96]	0.52
Both	5/32	0.53	[0.18–1.62]	0.27
Serum albumin level				
> 3.1 g/ml	10/68	1.00		
< 3.1 g/ml	18/61	2.43	[1.02–5.78]	0.05
Serum CRP level				
< 2.2 mg/dl	8/62	1.00		
> 2.2 mg/dl	20/67	2.87	[1.16–7.13]	0.02

*CI* confidence interval, *CRP* C-reactive protein, *ECOG PS* Eastern Cooperative Oncology Group performance status, *GEJ* gastroesophageal junction, *NC* not calculated, *OR* odds ratio, *SPM* severe peritoneal metastasis

## Discussion

This is the first study to compare the efficacy and safety of fluoropyrimidine with and without platinum as the first-line chemotherapy for AGC with SPM. This retrospective multicenter study suggested that combination therapy with fluoropyrimidine/platinum might be more effective than fluoropyrimidine monotherapy in terms of the OS, the PFS, and the improvement in both ascites and oral intake, associated with acceptable toxicity. Even considering the difference in patient background, multivariate analysis showed that both OS and PFS were significantly better in the FP group compared with the F group. We also determined significant risk factors for early death that are not likely to promote adaptation to chemotherapy among patients.

The general health condition of the subjects was poor in this study, as shown by the high proportion of patients with a high PS (40% had a PS > 2) and low serum albumin level (the median was 3.1 g/ml). This study also revealed treatment bias: monotherapy more likely to be selected for patients with worse general health statuses. Indeed, the F group included more elderly patients, more patients with a PS > 2, and more patients with ‘both’ SPM subtype. However, this mirrors actual clinical practice for the treatment of patients with AGC and SPM.

Of note, the F group showed comparable outcomes to those reported in previous studies. Iwasa et al. reported that the median TTF and OS were 1.9 and 4.6 months among 92 patients with AGC and SPM receiving 5-FU monotherapy regimens, including 44 (48%) with a PS > 2 [9]. Also, Hara et al. reported median PFS and OS of 2.4 and 6.0 months among 30 patients with AGC and SPM receiving 5-FU/*I*-LV, including 15 (50%) with a PS > 2 [10]. In our study, the median TTF, PFS, and OS were 1.4, 2.3, and 5.0 months, respectively, in the F group. Given that the median OS for patients with AGC receiving best supportive care (BSC) is about 3 months [15, 16], fluoropyrimidine monotherapy appeared to confer a modest survival benefit of about 2–3 months over BSC. In contrast, in the current study, the median OS in the FP group was 9.0 months, which may account for a minimum of 6-month survival benefit over BSC. We demonstrated that fluoropyrimidine/platinum combination therapy significantly prolonged OS and PFS compared with fluoropyrimidine alone, which was confirmed by the adjusted HR. This result is consistent with SPIRITS trial that demonstrated combination of S-1 plus cisplatin was superior to S-1 alone for the patients with general AGC. Furthermore, combination therapy with fluoropyrimidine/platinum induced a greater improvement in ascites and a greater improvement in oral intake. Such combination therapy could, therefore, be a promising treatment option for improving the prognosis and reducing the undesirable symptoms of AGC with SPM. Looking forward, we have planned further investigations to explore the most promising combination therapies based on fluoropyrimidine and platinum. This study and another small retrospective study of patients with AGC and SPM suggest that the FOLFOX regimen might be a good candidate [14].

Although there was a higher incidence of grade 3 or more neutropenia in the FP group, both FP and F were feasible. However, there was a high incidence of early death within 30 days of last administration (11% in the FP group and 25% in the F group), including too early death within 30 days of treatment start (2% in the FP group and 9% in the F group), because of rapid disease progression. This raises the issues of appropriate patient selection for chemotherapy and of the correct timing of switching to BSC, which require further investigation in future studies.

At present, prolongation of the survival of patients with AGC and SPM is highly warranted. Paclitaxel, which has been shown to exert activity on peritoneal metastasis, is another candidate to develop more effective chemotherapy regimens. When used as first-line chemotherapy, a randomized phase III trial (PHOENIX-GC trial) of patients with AGC and peritoneal metastasis showed that combination therapy with intravenous and intraperitoneal paclitaxel plus S-1 lengthened the OS, albeit without statistical significance, compared with S-1 plus cisplatin (HR, 0.72; 95% CI, 0.49–1.04; *p* = 0.081) [17]. When used as second-line chemotherapy, a randomized phase II trial (JCOG0407 trial) of patients with AGC and peritoneal metastasis refractory to fluoropyrimidine showed that weekly paclitaxel lengthened the PFS compared with the best available 5-FU regimen (HR, 0.58; 95% CI, 0.38–0.88; *p* = 0.005) [18]. A first-line combination strategy using paclitaxel has been developed for patients with SPM. The FLTAX regimen, which combines 5-FU/*l*-LV with paclitaxel, has had its recommended dose and feasibility confirmed for patients with AGC and SPM in a phase I trial [19]. Currently, a randomized phase II/III trial (JCOG1108/WJOG7312G trial: UMIN000010949) is ongoing to evaluate its efficacy and safety compared to 5-FU/*l*-LV, and this study will provide clinically important information. If the preliminary results can be confirmed, we will need to consider which of FP and FLTAX is more effective for the treatment of AGC with SPM.

To the best of our knowledge, few studies have predicted patients who would be adaptive to first-line chemotherapy in this patient group. To identify such patients, we explored risk factors for early death in this study. We determined early death to occur within 90 days after treatment initiation because this period of life expectancy is generally considered extremely short to initiate chemotherapy. We found two significant risk factors for early death: serum albumin level < 3.1 g/ml and CRP level > 2.2 mg/dl. Lower serum albumin level and higher serum CRP level are the possible indicators of poor patient condition and degree of disease aggressiveness. We suggest that these factors indicate extremely short life expectancy for chemotherapy, thus indicating that these patients should receive BSC. Future studies should expand on our exploratory findings.

This study has two key limitations. First, this was a retrospective study, and we could not exclude the possibility that the outcomes were affected by the different patient characteristics between treatment groups, despite adjustment for the most likely factors in the multivariate analysis. Therefore, these results should be carefully interpreted during consideration for incorporation into clinical practice. To resolve this critical issue, prospective studies are necessary. Second, the definition of SPM included heterogeneous subtypes, and it remains unclear whether the same treatment strategy is appropriate for all potential subtypes. Further discussion of this point is required if we are to develop meaningful treatment options for this population in the future.

## Conclusions

Fluoropyrimidine and platinum combination therapy might be more effective than fluoropyrimidine monotherapy for AGC patients with SPM. Further investigation is warranted for development of the treatment options in this population.

## Abbreviations

AGC: Advanced gastric cancer
OS: Overall survival
PFS: Progression-free survival
SPM: Severe peritoneal metastasis
TTF: Time to treatment failure
